# Length of the Remnant Cystic Duct and Bile Duct Stone Recurrence: a Case‒Control Study

**DOI:** 10.1007/s11605-023-05607-x

**Published:** 2023-03-01

**Authors:** Oliver Burckhardt, Sarah Peisl, Benoit Rouiller, Emilie Colinet, Bernhard Egger

**Affiliations:** 1 Department of Surgery, HFR Fribourg – Cantonal Hospital, Chemin Des Pensionnats 2-6, 1752 Villars-Sur-Glâne, Switzerland; 2 Department of Surgery, Valais Hospital, Sion, Switzerland; 3 Department of Radiology, HFR Fribourg – Cantonal Hospital, Villars-Sur-Glâne, Switzerland; 4 University of Fribourg, Av. de l’Europe 20, 1700 Fribourg, Switzerland

**Keywords:** Cholecystectomy, Cystic duct, Bile duct stone, Cholelithiasis

## Abstract

**Background:**

Since the introduction of the Critical View of Safety approach in laparoscopic cholecystectomy, exposure of the common bile duct, and common hepatic duct is not recommended, therefore, the length of the cystic duct remnant is no longer controlled. The aim of this case‒control study is to evaluate the relationship between the length of the cystic duct remnant and the risk for bile duct stone recurrence after cholecystectomy.

**Methods:**

All MRIs with dedicated sequences of the biliary tract taken between 2010 and 2020 from patients who underwent prior cholecystectomy were reviewed. The length of the cystic duct remnant was measured and compared between the patients with and without bile duct stones using multivariate logistic regression analysis.

**Results:**

A total of 362 patients were included in this study, 23.5% of whom had bile duct stones on MRI. The cystic duct remnant was significantly longer in the patients with stones than in the control group (median 31 mm versus 18 mm, *P* < 0.001). In the MRIs performed > 2 years after cholecystectomy, the cystic duct remnant was also significantly longer in the patients with bile duct stones (median 32 mm versus 21 mm, *P* < 0.001). A cystic duct remnant ≥ 15 mm in length increased the odds of stones (OR = 2.3, *P* = 0.001). Overall, the odds of bile duct stones increased with an increasing cystic duct remnant length (≥ 45 mm, OR = 5.0, *P* < 0.001).

**Conclusions:**

An excessive cystic duct remnant length increases the odds of recurrent bile duct stones after cholecystectomy.

## Introduction

Cholecystectomy is one of the most commonly performed abdominal surgeries worldwide, and the laparoscopic approach is currently considered the gold standard for the treatment of gallstone disease.^1^ As the rate of bile duct injury in the first years after the first laparoscopic cholecystectomy is increased compared to open cholecystectomy, the focus has shifted to preventing those injuries, leading to the adoption of the Critical View of Safety (CVS) routine.^2^ The aim of safe cholecystectomy principles is to promote the recognition of gallbladder elements before resection to reduce the risk of the common bile duct and vascular injuries and avoid mistakes due to anatomical alterations and altered visual perception.^3,4^ CVS is achieved by dissecting the entire infundibulum off the liver bed and exposing the elements of the Calot triangle before resection.^1,5,6^ This routine is currently widely used and recommended by a multisociety consensus conference on the prevention of bile duct injuries during cholecystectomy, which included the Society of American Gastrointestinal and Endoscopic Surgeons (SAGES), the American Hepato-Pancreato-Biliary Association, the International Hepato-Pancreato-Biliary Association, the Society for Surgery of the Alimentary Tract, and the European Association for Endoscopic Surgery.^5,7^ CVS is also the method recommended by the SAGES Safe Cholecystectomy program.^1^ This approach led to a significant reduction in the major bile duct injury rate after laparoscopic cholecystectomy to 0.15–0.36%.^7^

Even though the overall morbidity and mortality rates associated with this surgical procedure were reduced. ^6^ the issue of recurrent bile duct stones (BDSs) after cholecystectomy remained. The rate is estimated to be 1–3%^8^ and has been reported to be higher in patients undergoing laparoscopic cholecystectomy than in those undergoing open cholecystectomy.^9–11^ Recurrent BDS is associated with complications, such as pancreatitis and cholangitis, increasing long-term morbidity and mortality.^10^

The etiology of recurrent BDS has not yet been fully elucidated, but a possible association with the remnant cystic duct stump has been suggested.^9,11^ The first observation of a cystic duct remnant (CDR) with a so-called reformed gallbladder containing stones was published in 1912 by Flörcken,^12^ and in 1966, Bodvall et al. correlated the severity of postoperative biliary distress with the presence of a CDR measuring ≥ 10 mm in length.^13^ Subsequently, other authors have also reported this correlation, and they have reported significant CDR lengths of 0.5, 1, and 1.5 cm.^11,14–16^ However, very little is known about the CDR length after cholecystectomy, with one study from 1992 reporting a length < 1 cm in 34.5%, 1–2 cm in 36.3%, 2–3 cm in 24.8%, and > 3 cm in 4.4% of patients after laparoscopic cholecystectomy.^17^ Moreover, CVS does not take into account the CDR length because the focus on safe cholecystectomy principles lies in the prevention of bile duct injuries. Applying the CVS principles does not require exposure to the common bile duct and common hepatic duct.^18^

Considering the hypothesis that a longer cystic duct remnant increases the risk of bile duct stones and, thus, the risk of long-term postoperative complications, the aim of this study was to determine the relationship between cystic duct remnant length and the reappearance of bile duct stones after cholecystectomy.

## Materials and Methods

### Data Collection

This case‒control study was conducted in accordance with the STROBE criteria (http://strobe-statement.org). First, all abdominal MRIs (magnetic resonance imaging) performed between January 1, 2010, and December 31, 2020, on adult patients (> 18 years old) at our institution were screened, and patients with remaining gallbladder were excluded. After this initial screening, the MRIs were re-evaluated by a board-certified radiologist and excluded if there were no dedicated MRI sequences of the biliary tract; if a biliodigestive anastomosis was present; or bilio-pancreatic anatomical variations were present, such as periampullar diverticulum or a modified hilar anatomy. Patients presenting with periampullary diverticula were excluded due to the higher risk of recurrent BDS, especially after cholecystectomy,^19,20^ and patients with modified hilar anatomy, e.g., due to tumor infiltration, were excluded because of the potentially altered radiological interpretation.

For all included patients, the age at the time of MRI, sex, indication for MRI, and date of cholecystectomy were extracted from written radiological reports if available. The presence of gallstones in the biliary tract was evaluated, and the length of the CDR was measured by a board-certified radiologist on the cholangio-MRI 3D magnetic resonance cholangiopancreatography (MRCP) sequence (IRM 3 T GE) in the axial, coronal, or sagittal plane depending on the best visualization of the CDR. The maximal length measured for each patient was then extracted. The patients without stones were defined as the control group.

According to the literature, remnant BDS was defined as stones present on MRCP in the first 2 years after cholecystectomy, and recurrent BDS was defined as stones present on MRCP at least 2 years after cholecystectomy.^10,21–23^ Therefore, to evaluate the CDR length in BDS recurrence, a subgroup analysis with MRCP performed within and at least 2 years after cholecystectomy was performed.

### Statistical Analysis

Descriptive statistics were determined for the demographic data. Dichotomous data were reported as numbers and proportions, and continuous data were reported as the medians and interquartile ranges (IQRs). Demographic data of both groups were compared using logistic regression. Normal distribution was assessed with Shapiro‒Wilk’s method. The effect of CDR length on the presence of gallstones was analyzed using a multivariate logistic regression model adjusted for age, sex, and indication for MRI. The odds ratios (ORs) and 95% confidence intervals (CIs) were estimated. All statistical analyses were performed using R. A two-sided level of significance of 0.05 was utilized for all analyses.

## Results

### Patient Selection

**Fig. 1:**
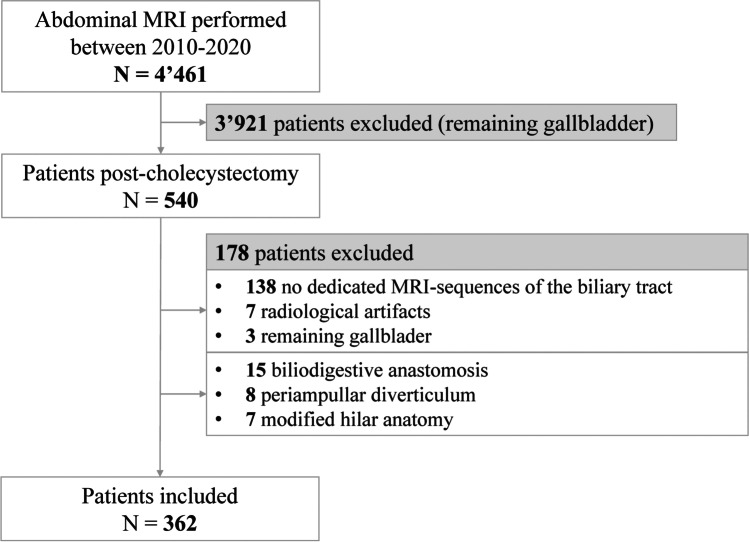
Flowchart of the patient selection

All radiological reports of the patients who underwent abdominal MRI at our institution from 2010 to 2020 (*N* = 4 461) were screened for a remaining gallbladder (Fig. 1). After screening, 540 MRI scans of the patients with a history of cholecystectomy were analyzed by a radiologist; 145 patients were excluded due to the absence of dedicated MRI sequences of the biliary tract or radiological artifacts. Three patients presented with a remaining gallbladder on radiological re-evaluation and were excluded. Among the remaining patients, 30 were excluded due to anatomical variations according to our exclusion criteria. Finally, 362 patients were included in the analysis.

### Baseline Characteristics

The baseline characteristics of the study population are provided in Table 1. In 256 patients (70.7%), MRCP was performed due to symptoms compatible with biliary stones, such as abdominal pain, cholestasis, dilatation of the biliary tract on other imaging, or pancreatitis and cholangitis. In 102 patients (28.2%), an abdominal MRI was performed for follow-up of a pathology that was not related to biliary stones, such as liver cirrhosis, cystic lesions of the pancreas, or inflammatory bowel disease. Overall, 85 patients (23.5%) presented with stones in the biliary tract on MRCP and were compared to the control group without stones (277 patients). Symptoms compatible with biliary stones were significantly more frequent in the patients with BDS on MRCP than in controls (*N* = 76, 89.4% vs. *N* = 180, 65.0%, *P* < 0.001). The patients presenting with stones were significantly older, with a median age of 69 compared to a median age of 63 years in the control group. No significant difference was found related to sex.

**Table 1:** Baseline characteristics of the study population

	Overall *N* = 362	BDS *N* = 85	No BDS (control group) *N* = 277	OR		95% CI	*P* value^a^
Sex, no. (%)							
Female	239 (66.0%)	55 (64.7%)	184 (66.4%)	1			
Male	123 (34.0%)	30 (35.3%)	93 (33.6%)	1.1		(0.64;1.78)	0.770
Age at diagnosis in years, median (IQR)	64 (50; 75)	69 (54; 78)	63 (49; 74)	-		-	0.011^b^
Indication for MRI, no. (%)							
Symptoms compatible with BDS^c^	256 (70.7%)	76 (89.4%)	180 (65.0%)	1			
Not related to biliary stones	102 (28.2%)	8 (9.4%)	94 (33.9%)	0.2		[0.09;0.41]	< 0.001
Unknown	4 (1.1%)	1 (1.2%)	3 (1.1%)	0.8		[0.04;6.28]	0.839
Interval between MRI and cholecystectomy, no. (%)							
> 2 years	204 (56.4%)	55 (64.7%)	149 (53.8%)	1			
≤ 2 years	99 (27.3%)	24 (28.2%)	75 (27.1%)	0.9		[0.49;1.50]	0.614
Unknown	59 (16.3%)	6 (7.1%)	53 (19.1%)	0.3		[0.11;0.70]	0.010
Presence of biliary stones	85 (23.5%)	-	-	-		-	-

^a^*P* values were assessed by logistic regression (reference: no BDS) or ^b^by the median test. ^c^Cholestasis, pancreatitis, cholangitis or abdominal pain.* BDS* biliary duct stones, *OR* odds ratio, *CI* confidence interval, *IQR* interquartile range

MRCP was performed within 2 years after cholecystectomy in 27.1% of the patients (*N* = 99) and in 56.4% of the patients after more than 2 years (*N* = 204). No significant difference in the occurrence of stones based on the timing of cholecystectomy prior to MRCP was shown.

### Length of the Cystic Duct Remnant

The cystic duct stump was significantly longer in the patients with stones in the biliary tract than in the control group (median 31 mm vs. 19 mm, *P* < 0.001; Table 2). A subgroup analysis of the patients who underwent MRCP more than 2 years after cholecystectomy revealed a significant difference in the median CDR length between the patients with and without BDS (32 mm vs. 21 mm, *P* < 0.001).

**Table 2:** Effect of cystic duct remnant length on the presence of bile duct stones (multivariate logistic regression model adjusted for sex, age, and indication for MRCP)

	BDS	No BDS (control group)	*P* value
Overall	*N* = 85	*N* = 277	
CDR length in mm, median (IQR)	31 (20;45)	19 (12;29)	< 0.001
Age in years, median (IQR)	69 (54;78)	63 (49;74)	0.010
Male, *n* (%)	30 (35.3%)	93 (33.6%)	0.997
Indication for MRI = symptoms compatible with BDS, *n* (%)	76 (89.4%)	180 (65.0%)	0.760
Interval between MRI and cholecystectomy ≤ 2 years	*N* = 24	*N* = 75	
CDR length in mm, median (IQR)	25 (13;39)	17 (12;30)	0.076
Age in years, median (IQR)	62 (48;75)	61 (40;73)	0.531
Male, *n* (%)	8 (33.3%)	29 (38.7%)	0.491
Indication for MRI = symptoms compatible with BDS, n (%)	22 (91.7%)	63 (84.0%)	0.992
Interval > 2 years	*N* = 55	*N* = 149	
CDR length in mm, median (IQR)	32 (25;47)	21 (21;29)	< 0.001
Age in years, median (IQR)	69 (58;79)	63 (51;74)	0.021
Male, *n* (%)	20 (36.4%)	45 (30.2%)	0.533
Indication for MRI = symptoms compatible with BDS, *n* (%)	49 (89.1%)	88 (59.1%)	0.378
Interval unknown	*N* = 6	*N* = 53	
CDR length in mm, median (IQR)	31 (23;45)	18 (9;27)	0.050
Age in years, median (IQR)	79 (66;81)	69 (54;75)	0.096
Male, *n* (%)	2 (33.3%)	19 (35.8%)	0.996
Indication for MRI = symptoms compatible with BDS, *n* (%)	5 (83.3%)	29 (54.7%)	0.996

*BDS* biliary duct stones, *CDR* cystic duct remnant, *MRCP* magnetic resonance cholangiopancreatography, *IQR* interquartile range

**Fig. 2:**
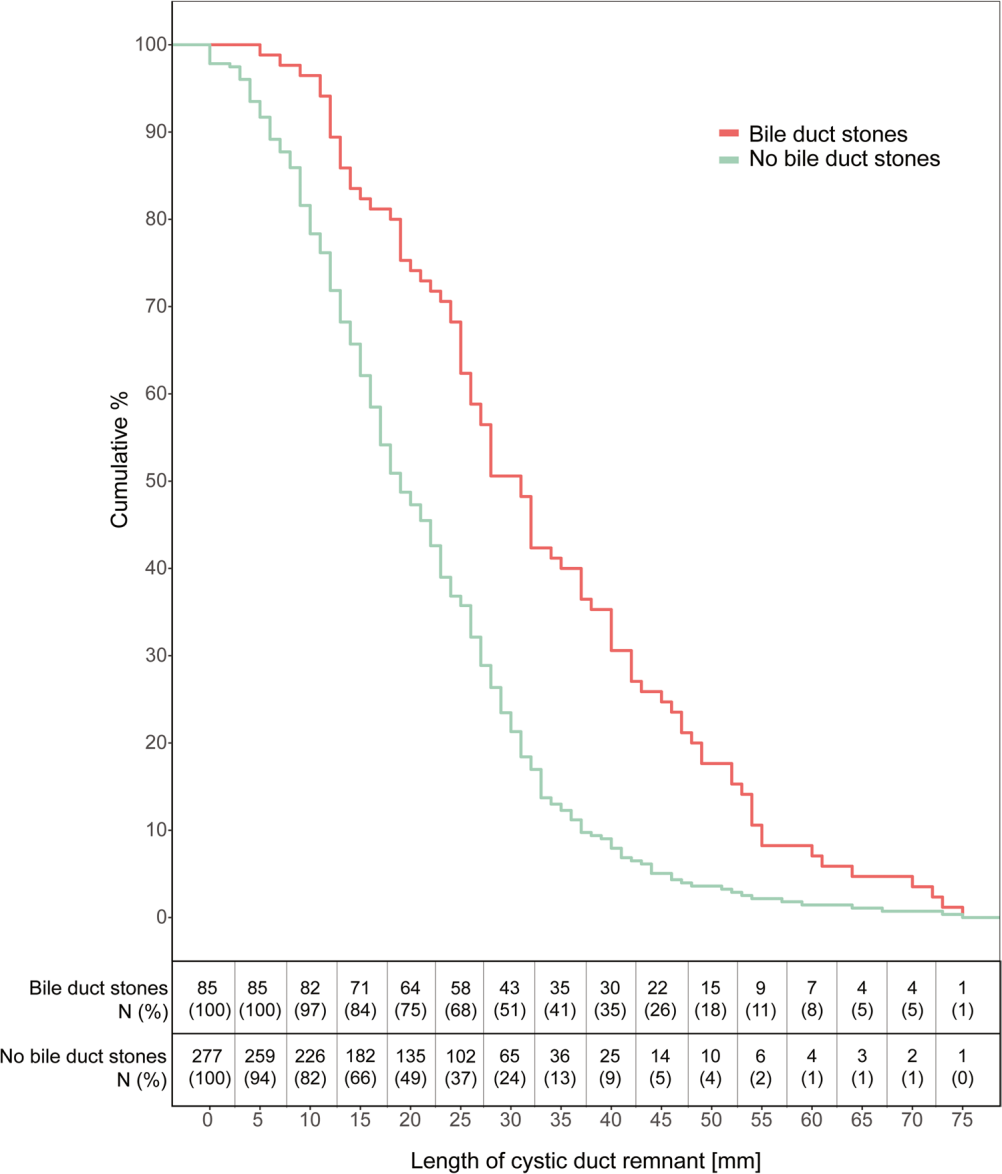
Cumulative percentage of the cystic duct remnant length in patients with and without bile duct stones on MRCP

**Fig. 3:**
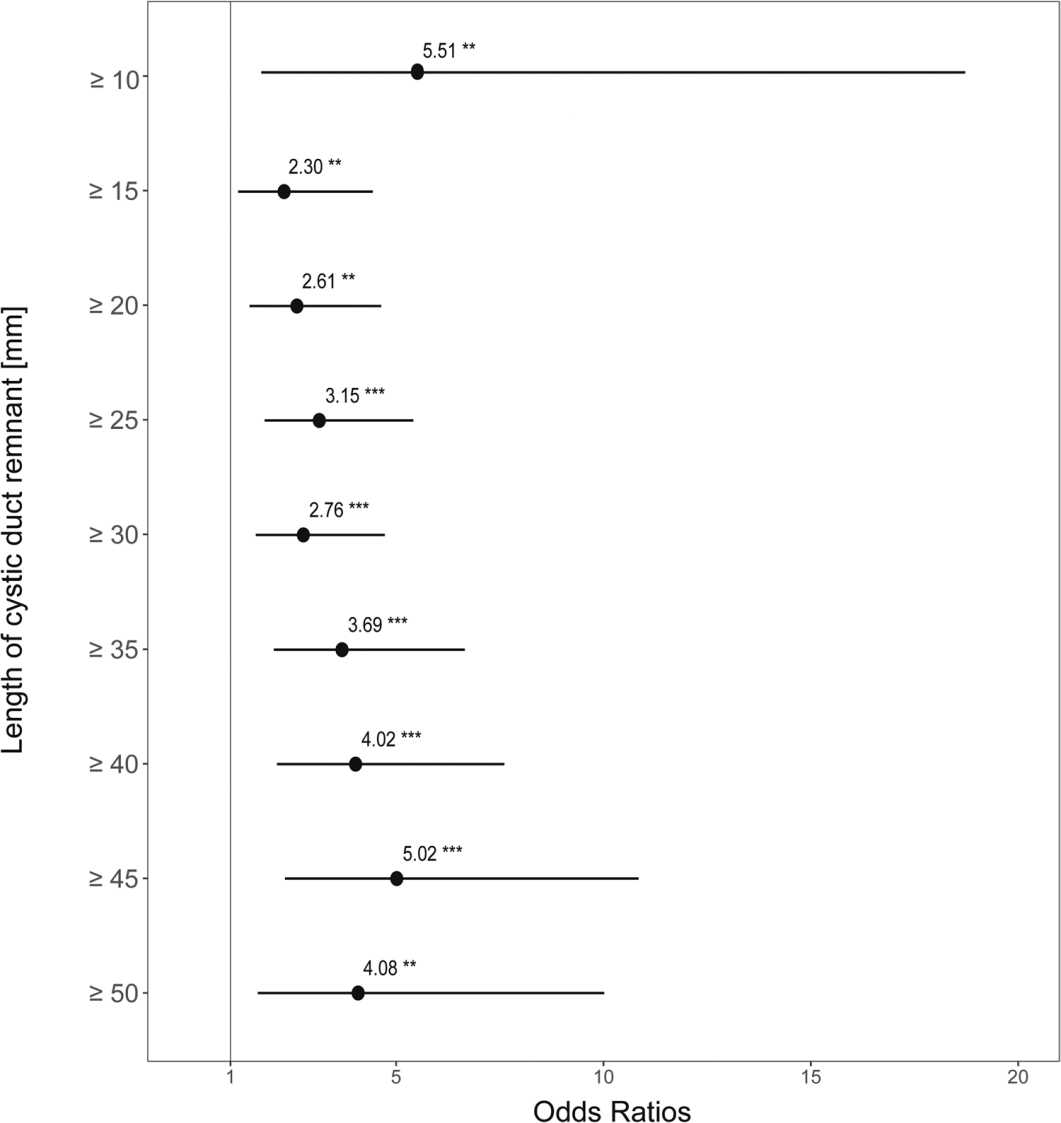
Forest plot of the cystic duct remnant length in patients with and without bile duct stones on MRCP. **P value < 0.005, ***P value < 0.001 (calculated by multivariate logistic regression, adjusted for age, sex, and indication for MRCP. Reference = no bile duct stones)

Figure 2 shows the cumulative curve of the patients presenting with and without BDS related to the CDR length. In a multivariate logistic regression model adjusted for age, sex, and indication for MRI (Fig. 3), a CDR ≥ 15 mm or ≥ 20 mm in length increased the odds of stones more than twofold (OR = 2.3, 95% CI 1.22;4.58, *P* = 0.001 and OR = 2.6, 95% CI 0.29;3.26, *P* = 0.001, respectively). Overall, the odds of BDS increased with increasing cystic duct stump length (CDR ≥ 45 mm, OR = 5.0, 95% CI 2.34;11.07, *P* < 0.001; Fig. 2).

## Discussion

Stones in the biliary tree are present in up to 1–3% of patients after cholecystectomy,^8,9^ and may lead to significant morbidity. Thus, our study aimed to evaluate whether the length of the cystic duct stump correlates with the occurrence of stones after cholecystectomy. Indeed, our data suggest that a long CDR is associated with a significant increase in BDS after cholecystectomy. In particular, the CDR was significantly longer in patients presenting with stone recurrence, i.e., at least 2 years after cholecystectomy.

Although a higher OR was calculated for a CDR > 10 mm in length, the large CI due to the small number of patients limits the statistical interpretation. Beyond 15 mm, however, every millimeter of remnant cystic duct increased the odds of gallstones. Few authors have previously described a possible correlation between symptoms following cholecystectomy and a CDR exceeding 10 mm in length.^13,14,24^ However, all studies were performed between 1966 and 1991 and included patients who underwent cholecystectomy before the laparoscopic era and the routine use of CVS.

To the best of our knowledge, this study is the first to describe the length of the CDR in the modern laparoscopic era. As described above, in the technique of laparoscopic cholecystectomy applying CVS, it is not recommended to obtain exposure of the entire cystic duct from the neck of the gallbladder to its union with the common bile duct; therefore, the length of the CDR remains unknown.

In our study, retrospectively measured cystic duct stumps showed that an increasing length was associated with higher rates of recurrent stones, suggesting that a shorter CDR might decrease BDS recurrence after cholecystectomy.

Our data do not question the validity of CVS. Considering the low risk of BDS recurrence and the high morbidity and mortality of bile duct injuries, aiming for a shorter cystic duct stump should not jeopardize safety. Thus, validated techniques to visualize the cystic duct and at the same time avoid bile duct injuries, such as transcystic intraoperative cholangiography^25–27^ or biliary tract visualization with near-infrared imaging with indocyanine green^28,29^ during cholecystectomy, might be employed to avoid exceedingly long CDR while maintaining the established safety principles.

The present retrospective, radiological study has several limitations. First, clinical data, especially regarding indication for surgery, open or laparoscopic approach, and other risk factors except for age and sex, were not available due to the study design. On the other hand, little is known about other factors associated with a higher risk of recurrent BDS after cholecystectomy, with most studies focusing on patients who have undergone endoscopic stone extraction with a preserved gallbladder. The known risk factors for gallstones include the composition and properties of the stones, female sex, age, and biological and lifestyle factors.^30,31^ Anatomical factors such as common bile duct angulation and initial common bile duct diameter have been described as potentially influencing the recurrence of stones after ERCP but have not been studied after cholecystectomy.^32,33^ More knowledge about the impact of these factors on recurrence rates after cholecystectomy is needed.

Furthermore, information about the preoperative presence of BDS was not available, raising the question of remnant BDS. To minimize this bias, a subgroup analysis of patients who underwent MRCP more than 2 years after cholecystectomy was performed. Whether patients underwent ERCP or spontaneous stone migration before MRCP was also rarely available. In addition, patients who underwent ERCP postoperatively without prior MRCP were not included. This probably led to an underestimation of the rate of symptomatic stones after cholecystectomy. However, our study showed a BDS frequency of 23.5% in patients for which an MRI had been prescribed, which is comparable with the study by Shiraz et al., who found that 17.6% of BDS in patients suffered from postcholecystectomy syndrome.^10^

Finally, only patients who had an MRCP after a cholecystectomy were included, leading to a selection bias with an overinclusion of patients with BDS (23.5%), although this complication is described in up to 1–3% of patients after cholecystectomy.^8,9^ However, adjusting the statistical model for the indication of the MRI did not show any difference.

The results of the present study are preliminary and warrant confirmation in a prospective study including further clinical data, broader risk stratification, and the use of systematic intraoperative cholangiography with measurements of the cystic duct.

## Conclusion

In conclusion, our results show a clear trend of longer cystic duct stump in patients presenting BDS and that every excess millimeter of CDR increases the odds of biliary stones after cholecystectomy. This suggests that a longer cystic duct stump may lead to increased morbidity and complications related to stones, such as cholangitis and pancreatitis. If technically feasible and under favorable anatomical conditions, aiming for a short CDR might be beneficial; however, the safety principles should by no means be neglected.

## Abbreviations

*CVS*: Critical View of Safety
*SAGES*: Society of American Gastrointestinal and Endoscopic Surgeons
*BDS*: Bile duct stone
*CDR*: Cystic duct remnant
*MRI*: Magnetic resonance imaging
*MRCP*: Magnetic resonance cholangiopancreatography
*IQR*: Interquartile range *OR*: odds ratio
OR: Odds ratio
*CI*: Confidence interval
